# Trial of an Internet‐Based Cataract and Lens Exchange Registry in a Tertiary Teaching Hospital: Insights and Impact on Clinical Workflow

**DOI:** 10.1155/joph/7251635

**Published:** 2026-04-03

**Authors:** Lauren Sartor, Chameen Samarawickrama

**Affiliations:** ^1^ Department of Ophthalmology, Westmead Hospital, Sydney, Australia, nsw.gov.au; ^2^ Translational Ocular Research and Immunology Consortium, Westmead Institute for Medical Research, Sydney, Australia; ^3^ Faculty of Medicine and Health, University of Sydney, Sydney, Australia, sydney.edu.au

## Abstract

**Purpose:**

To evaluate the feasibility of an internet‐based cataract surgical registry in a tertiary teaching hospital and compare the efficiency of a registry‐based, prospective cataract audit with retrospective methods.

**Setting:**

Westmead Hospital, Sydney, Australia.

**Design:**

A continuous time‐motion study.

**Methods:**

The duration of pre‐ and postoperative cataract consultations were measured before and after the introduction of the internet‐based Cataract & Lens Exchange Analysis & Register log. Outcomes assessed were the total duration of the consultation, medical record keeping and patient facing activities. A clinician survey was completed, and comparison was made in auditing times between the prospective registry data and the retrospective chart review.

**Results:**

Use of the registry added on average an extra 1.7 min to the standard consultation, but this was not significant (*p* = 0.95). Preoperative clinics (*p* < 0.01), language interpreter use (*p* = 0.05) and completion of consent forms (*p* = 0.04) lengthened the total duration of the consultation. Participants were positive about the benefit of the registry (85% positive) though there were mixed responses about the ease of use and integration of the registry in the existing workflow.

**Conclusion:**

The web‐based registry was successfully trialled in a tertiary hospital setting without adversely affecting consultation time. The registry offers a substantial time saving over retrospective audit methods, which has the potential to improve engagement in quality assurance activities.

## 1. Introduction

Clinical audits are an essential tool to the maintenance and delivery of high‐quality and cost‐effective healthcare services. They are essential to tracking clinical outcomes which can be used for benchmarking, [1], quality assurance and improvement activities [2]. Audits also have the benefit of revealing changes in patient risk factors, disease complexity [3, 4] and practice patterns [5] that may not be appreciated otherwise. One example is of a large retrospective clinical audit of over 5000 cataract cases undertaken at an Australian tertiary hospital [6], which identified a high prevalence of complex cataract cases (49%) when compared with previous audits and international benchmarks. This highlighted the local challenges that influence management and outcomes and was used as the foundation for improved service provision and funding.

In the context of cataract surgery, which is one of the most frequently performed elective procedures globally [7], retrospective audits can be onerous and time consuming. Furthermore, large audits can magnify the inherent disadvantages of retrospective methodology [8] including missing data [6, 9], bias in documentation and human error in data collection. These factors can compromise the quality of an audit and limit its findings, which ultimately affect the usefulness of the audit, patient outcomes and healthcare costs. Given the global scale of cataract surgery, there is a place for more robust and standardised methods of data collection [10] and prospective registries offer one solution.

Several large national and multinational cataract surgical registries have been successfully established [11–15] and have demonstrated their value in tracking changes in practice [16] and surgical outcomes [17]. Tertiary teaching hospitals are particularly well suited to registries for several reasons; they comprise rotating teams of trainees, see large volumes of clinically complex patients, have access to new therapies and can accommodate lengthy follow‐up periods. In centres such as these where there is variability in patient complexity and trainee experience, it is critical that clinical outcomes are evaluated to ensure the highest standard of care is delivered. Web‐based registries offer a solution for centres that do not have registries integrated with medical record–keeping software or use paper‐based documentation. In these contexts, a prospective web‐based registry has the potential to streamline and improve the quality of the audit process, in alignment with the national strategy [10]. However, despite these benefits, there are barriers to implementation of any new system in a healthcare setting [18, 19]. Studies of electronic medical record systems (EMRSs) have shown that perceived barriers to use include demands on clinician time [20] and disruption of clinical workflow [21]. In the context of a high volume clinic, a new registry could have real or perceived impacts on workflow and compromise direct patient‐facing care. Time‐motion studies (TMSs) are an ideal method to investigate the impact of a new registry on workflow [22].

The purpose of this TMS was to determine if a web‐based registry could be successfully implemented in a high‐volume tertiary outpatient ophthalmology clinic with minimal impact on patient flow. We also aimed to understand clinician attitudes for the registry and to determine if it would make the clinical audit process more efficient in the long run.

## 2. Methods

The study was a prospective, continuous, TMS conducted from August 2022 to June 2023 in the outpatient ophthalmology clinic at Westmead Hospital, a tertiary referral centre and university teaching hospital located in Sydney, Australia. It was approved by the Western Sydney Local Health District Human Research Ethics Committee (HREC 2021/STE01344) and followed the tenets of the Declaration of Helsinki.

The Ophthalmology Department comprises 28 specialist ophthalmologists and 5 accredited trainees that rotate at 3 monthly intervals and performs approximately 1600 cataract procedures annually [6]. Clinical audits are conduced quarterly as per the health service standards for continuous quality improvement. The audit process is retrospective and conducted by the junior medical officer (JMO) of the department, who is present for a 12‐month rotation. The quality and completeness of the audits are dependent on both the quality of the documentation and the skills and available time of the JMO conducting the audit, which can vary between audit cycles.

### 2.1. The Registry

The Cataract & Lens Exchange Analysis & Register log (CLEARlog; HOYA Surgical Optics) is an internet‐based registry that is accessible using an e‐mail login and password on any computer or tablet. It captures data pertaining to five stages of the cataract assessment and procedure and is divided into the following pages: ‘Patient information,’ ‘Presurgery,’ ‘Surgical plan,’ ‘Surgery’ and ‘Follow‐up.’ Each section is estimated to take 30–60 s to complete, varying with case complexity. A minimum of ‘presurgery’ and ‘follow‐up’ was entered, representing the clinical interactions encountered during the clinic.

### 2.2. Time and Motion Study

The model utilised a TMS [22, 23] design that has been used to evaluate both EMR systems in healthcare settings [24, 25] and ophthalmic registries [26]. This prospective TMS utilised the CLEARlog registry at routine patient presurgical and 1 month postsurgical appointments alongside the EMR system which is used for clinical record keeping (Cerner Millennium EMR platform [Cerner Corp., North Kansas City, MO]). The trainees were able to withdraw participation at any time or decline observation without withdrawing from the study. Trainees attended a group information session prior to implementation of the registry, where they were taken through the registry in a step‐by‐step tutorial and had the opportunity to use the registry and clarify questions. The registry was implemented with each group of trainees 6 weeks after they commenced the clinical term to allow them opportunity to familiarize themselves with the workflow of the clinic. The participating doctors had their consult times recorded both before and 4–6 weeks after implementation of the registry. Before eligible preoperative cataract clinics or individual postoperative appointments, the trainee was approached for consent to observation on that day. The observations took place over 2 clinical sessions on random days of the week. Observations of the consultations were completed by an observer (L.S) who was familiar with both the CLEARlog registry and the EMR.

To record the activities that took place during the consultations, a Microsoft Access tool designed for TMS was used [24]. The tool specifies 85 activities (e.g., talking with patient, examining patient and typing a note) that can take place during a consultation and lists them on a single screen. The observer selected the relevant activity from the list, with the start of a new activity indicating the end of the previous one. The activities were grouped into those affecting direct patient care (talking with patient and examination) and indirect patient care (reading medical record, typing notes, completing forms and reviewing imaging) and times were summed for analysis. Activities not related to patient care, i.e., answering phone calls or leaving the room during the consultation were excluded from analysis.

### 2.3. Registry Survey

All participating clinicians were asked to complete a deidentified survey (supporting document). The survey was adapted from one previously used to test the impact of an EMR [27] and clinical registry in healthcare settings [26], based on the Delone and McLean model of Information System success [28]. It consisted of 17 questions divided into 4 sections that tested the domains of: Information quality, System Quality, Satisfaction, Benefits. The questions were assessed on a 5‐point Likert scale of ‘agree,’ ‘somewhat agree,’ ‘neither agree nor disagree,’ ‘somewhat disagree’ and ‘disagree.’

### 2.4. Clinical Audit

The retrospective cataract audit was conducted as part of the routine quality improvement activities of the outpatient clinic. The audit was conducted for the preceding three months from data stored on the EMR system in the form of consult notes, biometry reports, operative reports and intraocular lens (IOLs) records. The information that is audited includes demographics (sex, age and laterality), ocular comorbidities, pre‐ and postoperative visual acuity, patient risk factors, the IOL used, intra‐ and postoperative complications and subjective refraction.

The steps of the audit process were as follows:

1. Log into the EMRS;
2. Generate a list of relevant medical record numbers (MRNs) through a search of operative procedures and refine the list to only include cataract surgery;
3. Access each relevant medical record and extract the data to a purpose built excel spreadsheet;
4. Clean the data to its appropriate format for analysis, including conversion of Snellen units to logMAR units;
5. Upload to statistical package (SPSS) and run analysis.

The overall time taken to conduct the retrospective audit and the time taken to review each patient file were recorded in minutes using the Microsoft access tool described above. The time taken to tabulate the results was not included in the analysis.

To conduct the audit using the web‐based CLEARlog registry, the following steps were taken:

1. Log into the web‐based registry and download the datafile;
2. Upload the file to the statistical package (SPSS) and run analysis

The overall time taken to conduct the audit using the CLEARlog cataract registry was recorded. The time taken to tabulate the results was not included in the analysis.

### 2.5. Statistical Analyses

The primary outcome measure was the total consultation time following implementation of the CLEARlog registry, measured in minutes. Secondary measures included time taken on indirect and direct patient care. Descriptive statistics were obtained for the retrospective audit times.

A power calculation was performed using the G‐Power application. A within‐factors one‐way ANOVA, with one group and two measurements calculated that a sample size of 65 observations would deliver a power of 0.8 and error of 0.05 and effect size of 0.05.

Statistical analyses were conducted using SPSS, with a *p* value of < 0.05 considered statistically significant. A mixed effects linear model was used to analyse the data, where the fixed effects that were controlled for were years of ophthalmology experience, type of cataract consultation (preoperative vs. postoperative), if a language interpreter was used, if consent forms were completed and if the CLEARlog registry was used. The random effect controlled for was the individual doctor identifier to account for the repeated measurements. This form of analysis was the most appropriate to use to account for the unequal number of pre‐ and postobservations including either or both interpreters and forms, with the preregistry observations serving as a control.

The survey responses for each item were tallied using SPSS and percentages calculated. The free‐text comments were grouped into common themes to facilitate qualitative interpretation.

## 3. Results

A total of 104 observations were recorded from 15 trainees (median level of ophthalmology experience 2 years (range: 1–4 years)) over February to November 2023. All doctors who were invited agreed to participate. Of the observations, 58 (56%) were preoperative and 46 (44%) were postoperative observations. The registry was utilised in 66 (64%) of the consultations and 12 (12%) of consultations utilised an interpreter.

### 3.1. TMS Outcomes

#### 3.1.1. Total Time

The mean total time for a preoperative appointment was 26.9 min (SD: 8.6 min), which was longer than the mean consultation time for postoperative appointments of 16.3 min (SD: 7.1 min; Table 1). The linear mixed‐effects model (Table 2) identified the significant effects of clinic type (preoperative clinics were a mean of 7.0 min longer than postoperative clinics, 95% CI: 2.4–11.6 min; *p* = 0.004), consent form completion (consent forms add a mean of 3.2 min onto the total time, 95% CI: 0.2–6.0 min; *p* = 0.035) and interpreter requirements (additional time of 4.6 min, 95% CI: 0.03–9.26; *p* = 0.049) on the total duration of the consultation. Training year (*p* = 0.62) and utilization of the registry (*p* = 0.95) did not have an effect on the total consultation time.

**TABLE 1:** Mean consultation times, reported in minutes.

	Clinic type	
	Preoperative (mins)	Postoperative (mins)
Total time	26.9 (8.6)	16.3 (7.1)
Interpreter required		
No	26.0 (8.6)	15.1 (6.2)
Yes	33.2 (5.4)	26.0 (6.9)
Forms to complete		
No	23.88 (8.8)	14.8 (7.7)
Yes	28.9 (8.0)	17.8 (6.2)
Registry utilised		
No	24.3 (7.1)	17.4 (8.5)
Yes	27.9 (9.0)	15.3 (5.5)

*Note:* Preoperative clinics were > 10 min longer than postoperative clinics.

**TABLE 2:** Summary statistics for fixed effects.

	Total time		Indirect care		Direct care	
	Change (mins)	95% CI	Change (mins)	95% CI	Change (mins)	95% CI
*Years exp*						
1 year	4.39	−3.05–11.84	3.53	−4.46–11.52	2.51	−2.00–7.04
2 years	2.00	−6.54–10.53	−0.20	−9.61–9.20	1.79	−3.39–6.98
3 years	1.17	−8.60–10.93	1.00	−9.69–11.68	−1.05	−7.12–5.02
4 years (ref)						

*Clinic type*						
Preop	7.01**^∗^**	**2.44**–**11.48**	0.46	−2.29–3.21	4.51**^∗^**	**1.74**–**7.27**
Postop (ref)						

*Interpreter required*						
Yes	4.64**^∗^**	**0.03**–**9.26**	0.63	−3.05–1.79	3.12**^∗^**	**0.31**–**5.93**
No (ref)						

*Consent forms*						
Yes	3.15**^∗^**	**0.22**–**6.09**	3.29**^∗^**	**1.82**–**4.77**	1.82**^∗^**	**0.02**–**3.62**
No (ref)						

*CLEARlog registry*						
Yes	−1.23	−2.89–3.08	0.84	−0.66–2.34	1.00	−3.06–0.60
No						

*Note:* Preoperative clinics were 7 min longer, interpreter requirement added almost 5 min and requirement for consent form completion added 3 min to the total consultation time. Utilization of the registry did not significantly affect total consultation time.

^∗^Significant results in bold.

#### 3.1.2. Indirect Care–Documentation, Consent Forms and Reading Time

When examining the outcome of indirect care time, only the completion of consent forms (additional 3.3 min, 95% CI: 1.8–4.8 min; *p* ∼0.001) had an effect on total consultation time. Training year (*p* = 0.686), clinic type (*p* = 0.741), registry utilization (*p* = 0.268; Figure 1(a)) and interpreter requirements (*p* = 0.608) did not affect indirect care.

FIGURE 1Boxplot of (a) indirect care and (b) direct care minutes for preoperative and postoperative clinics before and after registry implementation. Implementation of the registry did not significantly affect either direct or indirect care (*p* > 0.05).

**Figure:**
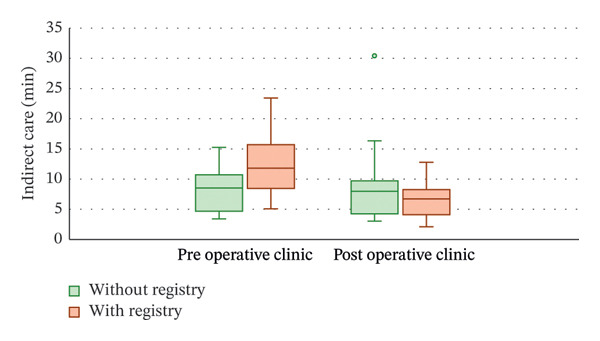
(a)

**Figure:**
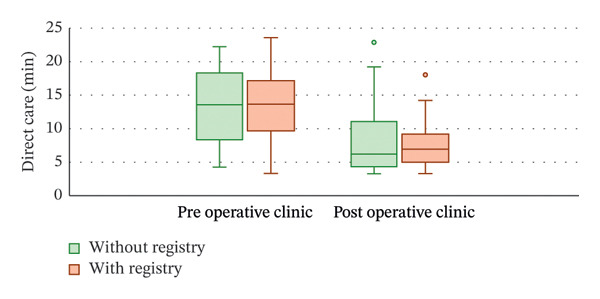
(b)

#### 3.1.3. Direct Care–Discussion and Examination

Time spent on direct care was affected by the type of clinic (preoperative appointments were 4.5 min longer, 95% CI: 1.7–7.3; *p* = 0.002), the presence of a language interpreter (adding 3.1 min, 95% CI: 0.3–5.9 min; *p* = 0.030) and consent form completion (adding 1.9 min, 95% CI: 0.02–3.6 min; *p* = 0.047). The training year (*p* = 0.394) and use of the registry (*p* = 0.184; Figure 1(b)) did not have an effect on the time taken for direct care.

### 3.2. Registry Survey

Thirteen of 15 participants completed the evaluation survey (87% response rate; Table 3). The overwhelming majority of participants were positive about the quality of information obtained through the use of the registry (94% positive response) and the quality of the registry system itself (92% positive response) as well as the benefit of the registry to patients, doctors and the hospital itself (85% positive response). However, there were mixed responses about the ease of use and integration of the registry in the existing workflow, with 48% having a positive opinion, 12% neutral and 40% negative opinion in that domain.

**TABLE 3:** CLEARlog evaluation survey responses for each item expressed in number and percentage.

Item	Agree *n* (%)	Somewhat agree *n* (%)	Neutral *n* (%)	Somewhat disagree *n* (%)	Disagree *n* (%)
Quality of information					
(1) It is easy to establish an understanding of the components of the registry	12 (92%)	1 (8%)			
(2) It is easy to find information in the registry	5 (38%)	4 (31%)	3 (23%)	1 (8%)	
(3) Information in the registry appears orderly and is easy to read	7 (54%)	6 (46%)			
(4) It is easy to document information in the registry	6 (46%)	7 (54%)			
(5) The information contained in the registry is relevant to cataract surgical procedures and associated patient care	11 (85%)	2 (15%)			
Quality of system					
(6) It has been easy for me to learn how to use the registry	13 (100%)				
(7) It has been easy to login to the registry	10 (77%)		3 (23%)		
(8) The registry responds satisfactorily when moving between screens	10 (77%)	3 (23%)			
(9) The registry is stable and does not crash	12 (92%)		1 (8%)		
Registry use					
(10) I find it easy to integrate use of the registry with my usual work procedures	1 (8%)	3 (23%)	1 (8%)	3 (23%)	5 (38%)
(11) Implementation of the registry entails new tasks for me	10 (77%)	3 (23%)			
(12) Generally, the registry has made my work easier			2 (15%)	7 (54%)	4 (31%)
(13) I would like to use the registry in future	4 (31%)	4 (31%)	3 (23%)		2 (15%)
Registry expectations					
(14) I expect the registry to benefit patients in the future	7 (54%)	4 (31%)	2 (15%)		
(15) I expect the registry to benefit trainees and consultants in the future	5 (38%)	4 (31%)	3 (23%)		1 (8%)
(16) I expect the registry to benefit the hospital in the future	8 (62%)	5 (38%)			

### 3.3. Retrospective and Registry Audit Time

The retrospective audit included 329 patients, with a mean (SD) audit time per patient of 5.8 (2.9) minutes. The total time taken to collect the audit data was 31.8 h, and the overall audit time was 34.4 h including data cleaning and analysis. The time taken to log into the CLEARlog registry and download the data was 0.8 min, with a total time of 16.3 min including data analysis.

### 3.4. Comparison of Time Between Retrospective and Registry Methods of Data Collection for Auditing

**FIGURE 2:**
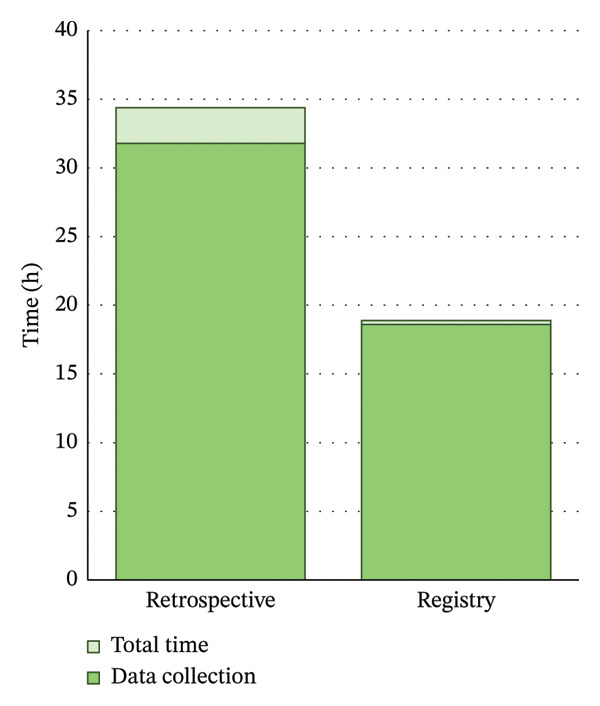
Comparison of time taken (in hours) to conduct retrospective and registry‐based audit. Use of the registry represented a saving of over 15 h per audit cycle.

The time taken to enter data into the registry during the consultation was a mean of 1.7 min (time per consultation), which equates to a total of 3.4 min to enter data per patient when accounting for both pre‐ and postoperative visits. This can be contrasted with the retrospective method of data collection, which takes a mean of 5.8 min to search each patient record from the EMR and transcribe both the pre‐ and postoperative data. Utilising the registry, the total data entry time is 18.6 h compared with the 31.8 h needed for retrospective data collection (Figure 2).

## 4. Discussion

This TMS demonstrates that a prospective, web‐based cataract registry can be implemented in a high‐volume, tertiary outpatient department with a minimal impact on consultation time, adding an average of 1.7 min to documentation time per patient visit without detracting from face‐to‐face patient care. The major benefit of the registry was the substantially improved efficiency in clinical auditing over retrospective methods, saving over 15 h per audit cycle. Furthermore, clinicians who utilised the registry overwhelming agreed that it would be of benefit to patients, doctors and the hospital, despite it adding to their clinical workload.

There is no doubt that clinical auditing is beneficial and should be encouraged, with impacts on the individual, the organisation and the broader profession. For the individual, audits provide a structured method to evaluate and improve personal clinical practice and are associated with an increased awareness of evidence‐based standards and improved clinical skills [29, 30]. At the organizational level, it supports quality assurance and improvement by systematically reviewing care delivery, benchmarking against standards and identifying areas for system‐wide change [31, 32]. As an example, a recent audit by our group identified the changing patterns of cataract referral to our institute, with a substantially increased prevalence of complex ocular pathology not documented previously, which has been used to change and improve the model of service delivery in our region [6]. On a professional scale, audit informs good governance and fosters the implementation of best practice [33]. Furthermore, large audits through clinical registries have been instrumental in guiding management, for example, in showing the benefits of intracameral antibiotics for the prevention of endophthalmitis [34]. As technology improves to facilitate auditing, it should become a part of the standard of care in all cataract centres. Registries have the potential to improve engagement with auditing by providing a user‐friendly platform and by improving the efficiency of the process in centres that rely on retrospective methods.

This study was also the first to examine the factors that influence cataract consultation length and complexity. Initial assessment clinics, completion of patient consent documentation and the use of a language interpreter add on average 7.1, 3.2 and 4.6 min, respectively, to the routine consultation time. Incorporating these findings into the structure of cataract clinics can improve and optimise the operational efficiency, especially in high‐volume clinics [35, 36], which in turn improves patient satisfaction and minimises clinician burnout [37]. Examples of successful integration include improving clinician‐patient ratios for time‐intensive initial assessment clinics, reducing in‐clinic waiting time by scheduling patient appointments according to consultation length [36], asking patients to prefill consent forms and medication lists before their appointment and anticipating the number of interpreter‐associated consultations and booking consultations accordingly.

Clinician burnout is an important consideration when introducing any new documentation task. Documentation takes a substantial amount of time for the clinician and increasing EMR demands have been linked to burnout [38, 39]. A registry platform that lies outside of an EMR system would require a clinician to duplicate data entry, once for official documentation and once for audit purposes. Even though the additional time required was on average only 1.7 min, this additional requirement was significant to many clinicians in our study as evidenced by the 52% who were neutral or negative about the integration of the registry into their workflow despite almost universal recognition of the benefits of the registry. Overcoming this “double data entry” challenge would be highly advantageous and would increase clinician acceptance of the registry. Innovative software has overcome this barrier, as seen with the IRIS and EURQUO [14, 40] cataract registries that effectively integrates with existing EMR systems [14], or EMR systems that integrates with existing registries (e.g., mediSIGHT EMR system integration into the National Ophthalmology Database [41]). Digital scribes are another tool that can be used to alleviate the burden of documentation and have been shown to reduce documentation time, cognitive load and burnout [42, 43]. They have the potential to shorten the cataract consultation time and efficiently record data for clinical and audit purposes. This is beyond the scope of this study but is an area for further development.

A limitation of this TMS was the difficulty in precisely recording the time taken for each activity due to the dynamic clinical environment clinicians work in. It was common for doctors to multitask during a consultation, such as discussing consent while completing paper‐based forms, and reflects the time pressured nature of high‐volume clinics. This limitation was addressed by using the overall consultation time as the primary outcome measure thereby capturing the entire clinical encounter. Additional factors that could bias the results include the speed of the computer processor, which varied with each observation and affected documentation times; the junior cohort of trainees who were skilled at navigating electronic software, which may not generalise to an older cohort; and the Hawthorne effect, where performance improves in the presence of an observer [23]. It is possible that the presence of an observer influenced trainees to engage with the program more than they would in other situations or that it has affected the overall consultation time. It was beyond the scope of this study to assess the quality of the data entered by the clinicians, and the survey was used to understand the trainee attitudes and likelihood of using the registry outside of the research context. The TMS would be strengthened by a larger cohort of participant clinicians with a wider range of experience, and a longer trial period. More generally, web‐based registries are also limited to settings with stable internet connectivity.

This TMS confirms that an internet‐based cataract registry can be successfully implemented in a busy tertiary outpatient department while maintaining an acceptable overall consult time. The internet‐based registry improved the efficiency of audits by over 15 h per audit cycle, making it an invaluable quality improvement tool. This is one of the first studies to examine the factors that lengthen a standard cataract consultation, which can be used to improve the efficiency of clinics. In the context of the ‘big data’ era [44], where continuous auditing is becoming a standard that ensures the quality and safety of clinical care, there is a place for internet‐based registries to fill a gap where prospective EMR‐based auditing tools are yet to be established.

## Funding

One author is a recipient of University of Sydney RTP scholarship and stipend from HOYA optics. One author is a recipient of a National Health and Medical Research Council Investigator Grant (APP:1175949).

## Disclosure

The sponsor and funding organisations had no role in the design or conduct of this research.

## Conflicts of Interest

The authors declare no conflicts of interest.

## Supporting Information

Supporting Information file contains clinician survey results.

## Supporting information


**Supporting Information** Additional supporting information can be found online in the Supporting Information section.

## Data Availability

The data that support the findings of this study are available from the corresponding author upon reasonable request.
