# Supplementary material for: Trial of an Internet‐Based Cataract and Lens Exchange Registry in a Tertiary Teaching Hospital: Insights and Impact on Clinical Workflow
Source: J Ophthalmol. 2026 Apr 3;2026:7251635. doi: 10.1155/joph/7251635 (PMC13051833; doi:10.1155/joph/7251635)
Supplement: Supplementary file 1 — Supporting Information Additional supporting information can be found online in the Supporting Information section. [file JOPH-2026-7251635-s001.docx]

**Supplementary Documents**

Evaluative Survey 2

**Evaluation of the Cataract & Lens Exchange Analysis & Register Log**

Part A

1. What is your current year of postgraduate training?

Part B – Quality of information

1. It is easy to establish an understanding of the components of the registry

| Agree  □ | Somewhat agree  □ | Neither agree nor disagree  □ | Somewhat disagree  □ | Disagree  □ |
| --- | --- | --- | --- | --- |

1. It is easy to find information in the registry

| Agree  □ | Somewhat agree  □ | Neither agree nor disagree  □ | Somewhat disagree  □ | Disagree  □ |
| --- | --- | --- | --- | --- |

1. Information in the registry appears orderly and is easy to read

| Agree  □ | Somewhat agree  □ | Neither agree nor disagree  □ | Somewhat disagree  □ | Disagree  □ |
| --- | --- | --- | --- | --- |

1. It is easy to document information in the registry

| Agree  □ | Somewhat agree  □ | Neither agree nor disagree  □ | Somewhat disagree  □ | Disagree  □ |
| --- | --- | --- | --- | --- |

1. The information contained in the registry is relevant to cataract surgical procedures and associated patient care

| Agree  □ | Somewhat agree  □ | Neither agree nor disagree  □ | Somewhat disagree  □ | Disagree  □ |
| --- | --- | --- | --- | --- |

Comments:

Part C – Quality of the system

1. It has been easy for me to learn how to use the registry

| Agree  □ | Somewhat agree  □ | Neither agree nor disagree  □ | Somewhat disagree  □ | Disagree  □ |
| --- | --- | --- | --- | --- |

1. It has been easy to login to the registry

| Agree  □ | Somewhat agree  □ | Neither agree nor disagree  □ | Somewhat disagree  □ | Disagree  □ |
| --- | --- | --- | --- | --- |

1. The registry responds satisfactorily when moving between screens

| Agree  □ | Somewhat agree  □ | Neither agree nor disagree  □ | Somewhat disagree  □ | Disagree  □ |
| --- | --- | --- | --- | --- |

1. The registry is stable and does not crash

| Agree  □ | Somewhat agree  □ | Neither agree nor disagree  □ | Somewhat disagree  □ | Disagree  □ |
| --- | --- | --- | --- | --- |

Comments:

Part D – Use

1. I find it easy to integrate use of the registry with my usual work procedures

| Agree  □ | Somewhat agree  □ | Neither agree nor disagree  □ | Somewhat disagree  □ | Disagree  □ |
| --- | --- | --- | --- | --- |

1. Implementation of the registry entails new tasks for me

| Agree  □ | Somewhat agree  □ | Neither agree nor disagree  □ | Somewhat disagree  □ | Disagree  □ |
| --- | --- | --- | --- | --- |

1. Generally, the registry has made my work easier

| Agree  □ | Somewhat agree  □ | Neither agree nor disagree  □ | Somewhat disagree  □ | Disagree  □ |
| --- | --- | --- | --- | --- |

1. I would like to use the registry in future

| Agree  □ | Somewhat agree  □ | Neither agree nor disagree  □ | Somewhat disagree  □ | Disagree  □ |
| --- | --- | --- | --- | --- |

Comments:

Part E – Expectations

1. I expect the registry to benefit patients in the future

| Agree  □ | Somewhat agree  □ | Neither agree nor disagree  □ | Somewhat disagree  □ | Disagree  □ |
| --- | --- | --- | --- | --- |

1. I expect the registry to benefit trainees and consultants in the future

| Agree  □ | Somewhat agree  □ | Neither agree nor disagree  □ | Somewhat disagree  □ | Disagree  □ |
| --- | --- | --- | --- | --- |

1. I expect the registry to benefit the hospital in the future

| Agree  □ | Somewhat agree  □ | Neither agree nor disagree  □ | Somewhat disagree  □ | Disagree  □ |
| --- | --- | --- | --- | --- |

Comments:
